# New insight into the role of MDMX in MDM2-mediated p53 degradation and anti-cancer drug development

**DOI:** 10.18632/oncoscience.542

**Published:** 2021-08-09

**Authors:** Jing Yang, Yanping Zhang

**Affiliations:** ^1^Jiangsu Province Key Laboratory of Immunity and Metabolism, Xuzhou Medical University, Xuzhou, Jiangsu, China; ^2^Department of Pathogenic Biology and Immunology, Xuzhou Medical University, Xuzhou, Jiangsu, China; ^3^Department of Radiation Oncology and Lineberger Comprehensive Cancer Center, School of Medicine, University of North Carolina at Chapel Hill, Chapel Hill, North Carolina, USA

**Keywords:** MDMX, MDM2, p53

## Abstract

Inactivation of the tumor suppressor p53 has been generally accepted as a hallmark of tumor. MDM2 and MDMX, the two closely related proteins are considered to be critical for negatively regulating p53 activity through inhibitory binding to and post-translational modification of the p53 protein. We have demonstrated that MDMX facilitates MDM2-mediated p53 ubiquitination and degradation via recruitment of the ubiquitin-conjugating enzyme UbcH5c to the MDM2-MDMX heterooligomers. Here, we discuss our new findings from genetically engineered mouse models and a potential therapeutic strategy.

## INTRODUCTION

The p53 stress response is an important regulator of cellular homeostasis, responding to a variety of stressors and eliciting hallmark effects on cell proliferation, apoptosis, and senescence [1]. As such, p53 plays an essential role in regulating the balance between cellular growth, proliferation, and destruction. Disturbance of this balance through p53 mutations leads to a wide variety of cancers as well as many other diseases [2]. Due to the high rate of p53 mutation, deletion, and misregulation in human cancers, in theory p53 regulated signaling pathways should be attractive drug targets. However, effective therapeutics utilizing such pathways has yet to find their ways into the clinic [3–6]. The lack of a complete understanding of p53 regulation, particularly its *in vivo* regulation, is at least in part to blame. Most p53-activation compounds developed to date offer relatively specific MDM2 inhibition by targeting the p53-binding domain on MDM2, such as nutlin [7]. However, their efficacy in patients has been disappointing, which can be attributed to multiple factors and the most prominent one is the on-target toxicity caused by exaggerated activation of p53 [8]. However, even if side effects could be minimized, it is possible that MDM2 inhibition alone could be ineffective because inhibitors that target only MDM2 and not MDMX are ineffective against cancer cells that overexpress MDMX. Thus, although potent MDM2 inhibitors are currently available, they are more successful in laboratory research than in clinic. In addition to preventing interaction with p53, other methods of inhibiting MDM2 and MDMX include the inhibition of MDM2 E3 ligase activity or the MDM2-MDMX heterooligomerization. However, studies have suggested that the MDM2-MDMX RING-RING oligomer is not an attractive drug target due to the lack of a defined catalytic site and deep pocket for small compound insertion [9, 10]. Drugs that can target MDM2 E3 ligase activity and simultaneously inhibit MDMX to maximize p53 stabilization and activation are clinically desirable.

Recent studies from our lab and others’ have demonstrated that MDM2 and MDMX heterooligomerization through their RING domains is essential for p53 control, while the MDM2 E3 ligase function is dispensable for growth and development under unstressed conditions but becomes essential for survival after genotoxic stresses [11–13]. While the current models suggest that MDM2 and MDMX function through both independent and interdependent pathways to regulate p53, and both MDM2 and MDMX are essential in keeping p53 activities in check, these two homologues are also interact with and regulate one another. *In vitro* experiments have demonstrated a greater affinity for the formation of MDM2-MDMX heterooligomers than either MDM2 or MDMX homooligomers alone [14]. A proposed function for the MDM2-MDMX heterooligomer posits that it results in preferential degradation of MDMX and stabilization of MDM2 [15–17]. Studies have also shown that the MDM2-MDMX heterooligomer works as a better E3 ubiquitin ligase than MDM2 homooligomer alone, suggesting that MDMX facilitates MDM2 E3 function [18]. Consistent with this notion, ectopic overexpression of MDMX enhances MDM2 E3 ligase activity toward p53 [19] and rescues the activity of certain MDM2 mutants lacking E3 ligase activity [20]. However, despite intense research exactly how MDM2 and MDMX work individually and together to regulate p53 is still partially understood, and sometime striking differences can exist between *in vitro* and *in vivo* studies.

A major obstacle of investigating individual functions of MDM2 and MDMX in mice is the early embryonic lethality caused by inactivation of either one while in the presence of p53. To solve this problem we took advantage of a previously developed system [21], in which the p53 gene is replaced by one encoding a fusion protein containing a full-length p53 fused C-terminally with the hormone-binding domain of a modified estrogen receptor (p53^ER^). The *p53^ER^* gene is expressed at physiological levels under control of the native promoter [22]. The p53^ER^ activity can be rapidly switched between WT and knockout states by administration and withdrawal of 4-hydroxytamoxifen (4-OHT). We generated mice expressing inducible *p53^ER^* under various MDM2 and MDMX deletion and mutation backgrounds and studied p53 regulation in mice and MEF cells. Unexpectedly, using the inducible p53^ER^ system we found that *in vivo* MDMX is essential for p53 degradation, as we found that in the presence of WT MDM2 and absence of MDMX the degradation of p53 is hardly detected [23]. Several possible mechanisms can be attributed to the role of MDMX in MDM2 degradation of p53. For example, we found that while *in vivo* MDM2 and MDMX can interact with p53 in the absence of each other, but they bind p53 more efficiently as a heterodimer; and that the MDM2-MDMX heterodimerization promotes p53 cytoplasmic localization, where p53 is degraded by the cytoplasmic proteasome. However, we believe the most likely mechanism for MDMX to enable MDM2 E3 ligase activity is its ability to bind UbcH5c and bring it to MDM2 proximity. Previous studies have shown that knockdown UbcH5c in MCF-7 cells increases p53 expression [24], suggesting that UbcH5c is a decisive ubiquitin-conjugating enzyme for p53 degradation. Using the inducible p53^ER^ system, we found that MDMX, but not MDM2, interacts with UbcH5c, and that the ability of MDMX to interact with both UbcH5c and MDM2 is essential for MDM2 mediated p53 degradation [23]. We further demonstrated that the last seven residues of MDMX are required for UbcH5c binding, and that replacing MDM2 C-terminal seven residues with the MDMX C-terminal seven residues enabled MDM2 to interact with UbcH5c and enhanced MDM2 E3 ligase activity for p53 degradation in the absence of MDMX [23]. Based on this finding, targeting UbcH5c-MDMX binding should affect MDM2 E3 activity but not MDM2-MDMX interaction. Hence, in theory strategies or small molecule compounds that can disrupt UbcH5c-MDMX binding but not MDM2-MDMX binding would stabilize p53 but not activate it, since the MDM2-MDMX heterooligomer can bind to and suppress the transcriptional function of p53 without necessarily degrade it. Such UbcH5c-MDMX binding-targeting compounds can avoid exaggerated p53 activation caused by disrupting MDM2-p53 binding, but they may be used as p53 sensitizers in combination with chemo- and radio-therapeutics to activate p53 in a coordinated and controllable manner.
